# ROSA: Resource-Oriented Service Management Schemes for Web of Things in a Smart Home

**DOI:** 10.3390/s17102159

**Published:** 2017-09-21

**Authors:** Chun-Feng Liao, Peng-Yu Chen

**Affiliations:** 1 Department of Computer Science, National Chengchi University, Taipei 11605, Taiwan; 2 Program in Digital Content and Technologies, National Chengchi University, Taipei 11605, Taiwan; 3 StarVedia Corporation Inc., Zhubei 302, Taiwan; fulhanwuway@gmail.com

**Keywords:** IoT, Web of Things, REST, Web Services, Universal Plug and Play (UPnP), smart home

## Abstract

A Pervasive-computing-enriched smart home environment, which contains many embedded and tiny intelligent devices and sensors coordinated by service management mechanisms, is capable of anticipating intentions of occupants and providing appropriate services accordingly. Although there are a wealth of research achievements in recent years, the degree of market acceptance is still low. The main reason is that most of the devices and services in such environments depend on particular platform or technology, making it hard to develop an application by composing the devices or services. Meanwhile, the concept of Web of Things (WoT) is becoming popular recently. Based on WoT, the developers can build applications based on popular web tools or technologies. Consequently, the objective of this paper is to propose a set of novel WoT-driven plug-and-play service management schemes for a smart home called Resource-Oriented Service Administration (ROSA). We have implemented an application prototype, and experiments are performed to show the effectiveness of the proposed approach. The results of this research can be a foundation for realizing the vision of “end user programmable smart environments”.

## 1. Introduction

Although there is a rich output in the study of the smart home recently, its market acceptance is still low in practice. Many scholars point out the main reason is that most of the devices rely on a specific platform or technology, resulting in difficulties developing the application services [1,2]. In light of the success and popularity of Web technology, many researchers began to learn from the concept of Mashup emerged from Web 2.0 [3] to break through this difficult situation. The so-called Mashup refers to the concept of having developers compose the existing and reusable services together promptly using generally available and standardized Web techniques such as HTTP, HTML, JavaScript, XML or JSON to shorten the application development cycle [4]. For example, if we develop a food review guided tour application, through Google Map Application Program Interface (API), Facebook Graph API, and by having the open data APIs of delicacies as the data source, the key software components and the main source of information can be instantly obtained without having to construct the whole application from scratch. The unit of the composable Web-based API is usually called a Web Service, which emulates a remote invocation by utilizing an HTTP-based request-response message delivery mechanism. The main benefit of the Web Service is that when implementing the services, the developers can reuse the standardized, platform-independent, and the available HTTP clients and servers.

The architectural styles of the Web Services fall into two categories: WS-* style and RESTful style. The WS-* style Web Services use HTTP as the transportation medium, where the Remote Procedure Call (RPC) signatures (name, parameter, and type of a remote function) were wrapped in the body of an HTTP message [5]. A series of related specifications, with “WS-” as their beginning, have been rapidly developed in the past decade. The WS-* style Web Services are also known as the “Big” Web Services [6] in the sense that when compared to the RESTful Web Services, the stack of specifications for the WS-* style Web Services is large. Roy Fielding, a member of the HTTP standard committee, pointed out that the reason that the Web possesses a high degree of flexibility and scalability is that a specific network-based architectural style is taken into consideration while designing HTTP [7]. While the WS-* style Web Services only indicate method signature and its call through HTTP that carries messages with a specific format. It is technically equivalent to redefining the heavy-weight distributed object with HTTP and XML. It turns out to be that “The Web is simple”, yet “Big Web Services are not simple [8]”. Thus, to truly enjoy the benefit of Web architecture, we should see HTTP as an architectural style, rather than a transportation mechanism, which is called REST(REpresentation State Transfer) architecture [7] and Web services that are designed following REST architecture style is also known as RESTful Web services [9].

In the REST architecture, every service is seen as a resource, and each resource is identified by an URL. A Web client can access the resources through methods specified by HTTP (e.g., GET, POST, PUT, DELETE, HEAD, and OPTIONS) From the perspective of object-oriented design (OOD), its effect is equivalent to drawing up a unified interface for the resources. Thus, according to the Polymorphism principle of OOD, Web clients can access all resources by following the same set of HTTP methods. In this way, RESTful Web Services can be fully compatible with any Web clients. On this ground, Webber et al. pointed out that RESTful architecture possesses features which include the support of existing mainstream Web technology, scalability, and efficiency that is higher than WS-*, low coupling, interface uniformity and simplicity [10]. The survey from the Gartner Institute also shows that the amount of RESTful Web Services has increased significantly since 2008 [11]. After analyzing the important Web Services distribution sites ProgrammableWeb.com, researchers even found out the proportion of Web Services that adopted the REST architecture are as high as 75% in 2010, while the WS-* only accounts for 16% [12]. Consequently, REST has become the mainstream architecture for the contemporary industries that develop Web Services [13].

Nowadays, HTTP servers have been able to operate with memory less than 8 KB [14]. In other words, it is possible to embed components capable of handling HTTP requests into various kinds of appliances and sensor modules in the way of low cost. Thus, WoT has now become a considerably viable concept. Given the technology trend mentioned above and the increasing attention of REST architecture, the idea of Web of Things (WoT) arises from the research domain of pervasive environments such as a smart home [13]. In this paper, the term WoT refers to interconnected Web Things where a Web Thing refers to the software or hardware components that meet the Web Things requirement Level 0 (or more) specified in the Web Thing Model, a W3C member submission [15]. The benefits of applying the WoT concept into a pervasive environment are listed below:

1. Enables service Mashups: By using Web techniques, developers can access sensor modules or control the surroundings without having to spend a lot of time learning and integrating all kinds of vendor-dependent architectures or communication protocols in the home network [16,17]. As reported in [2], it is quite difficult to coordinate all kinds of smart devices to perform services in the contemporary smart environment and the greatest benefit of WoT is to develop such services using the service Mashup approach through the standard Web interfaces.
2. Eases loading of Web Things: To understand the overall context of client/server interactions in a Web application, it is also desirable to keep the session states. As noted by Fielding et al., HTTP is a stateless client/server protocol in the sense that “each request from client to server must contain all of the information necessary to under stand the request. [18]” In practice, many traditional Web applications keep session states on the server side, causing the heavy loading of the servers. Thus, Fielding et al. also pointed out that, in REST architecture, the session states should not be stored on the server and thus the states are kept entirely on the client side [18]. This feature happens to be appropriate to the pervasive environment: the computing and storage capabilities of the client (such as Home Gateway or user’s handset) that is used to deploy the applications, are usually more powerful than that of the server side (the Web things such as the embedded sensor modules or smart appliances).
3. Promotes flexibility of applications: As mentioned, while the session states are maintained at the Web clients, if the Web Thing B joins after the Web Thing A, which is the same function, disappeared, the state of its existence will not be affected for the client. Besides, the resource A can be used to substitute the failed B, given that these two different resources produce the same results. Note that deciding if two resources are equivalent is not a non-trivial task and is out of the scope of this paper. More discussions on this limitation is taken up in Section 6.4.
4. Makes the environment programmable via Web technologies: Previously, the barriers of creating services in a pervasive environment are high because of many existing and incompatible standards (e.g., X 10, Jini, and LonWorks). Nowadays, most developers are familiar with the Web technologies such as HTTP, JavaScript, Ajax, JSON, and XML. In a WoT-enabled pervasive environment, all Web Things can be accessed and managed programmatically via existing well-founded Web technologies.

In the context of a smart home, several research works have been proposed to support WoT at home. The core idea is to transfer all kinds of sensors, software services, and home appliances into the resources in the REST architecture, and thus they become Web things [19,20]. Moreover, 6LoWPAN [21] (IPv6 over Low-Power Wireless Personal Area Networks) technologies, which enables the sensors located at low power and lossy wireless networks such as 802.15.4 and BLE expose themselves as IPv6 nodes, make these sensors directly accessible via edge routers. The study of applications and systems based on CoAP (Constrained Application Protocol) [22], a 6LoWPAN-compatible and UDP-based application layer protocol that mimics the REST design principles, also becomes an active body of research [23,24].

**Figure 1:**
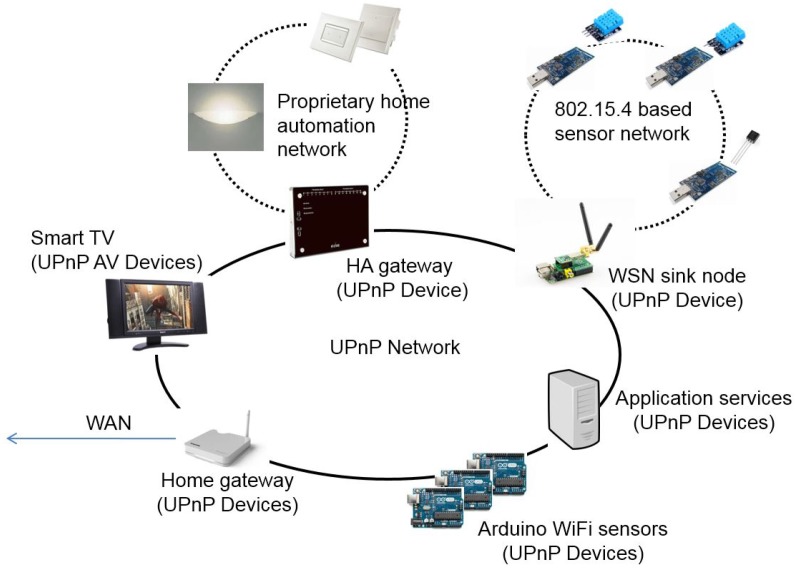
A UPnP-based home network.

**Figure 2:**
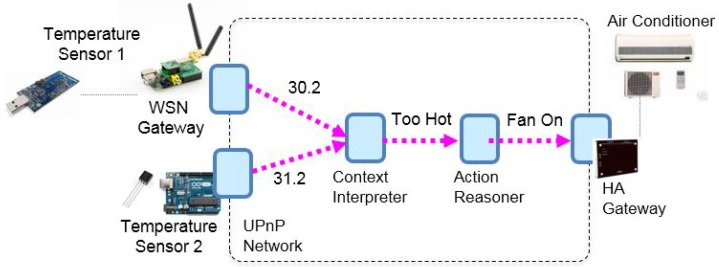
An air conditioning service in a UPnP-based home network. The dotted lines with arrowhead indicate logical data flows, which are mediated by a service manager under the hood.

However, as observed by Mathew et al. [25], several issues remain unsolved so far when applying WoT in a smart home. To make it clear, consider an automatic air conditioning service in a Universal Plug and Play (UPnP) [26] network (see Figure 1). The UPnP network consists of interconnected UPnP-enabled devices in an IP-based local area network. The devices located at non-IP networks can be accessed through protocol-specific gateways. For instance, the sensors in the ZigBee [27] network and the home appliances in the proprietary home automation network can be accessed respectively using the HA gateway and the WSN (wireless sensor network) coordinator. The air conditioning service (shown in Figure 2) is composed and maintained by an air conditioning service manager. To conduct a service composition, the service manager has to be aware of peer Web Things on the same home network (presence management). Then, it has to find the things that fit the needs of the air conditioning service by querying what these things can do and how to access them (service description and discovery). As shown in Figure 2, in the air conditioning service, temperature sensors, air conditioners, and the software components that reason about feelings and desired action is required. Finally, the service can be carried out by a sequence of remote invocations from the service manager to these “things” (invocation). Sometimes, the service manager registers for interesting events on the things so that when the registered events happen, the service manager can be notified (eventing). It can be observed from the example that the mechanisms mentioned above are fundamental to service composition. The technologies that are critical to support service composition (presence management, service description, and discovery, invocation and eventing) are collectively called “service management” in this paper.

Currently, little research works deal with WoT-based service management issues in a smart home. Taking service discovery as an example, most of the existing solutions adopt the centralized service directory architecture [23,28]. While the centralized directory is easy to implement and does not need to deal with the consistency problem, it requires to set up a dedicated and permanently available directory server, which results in high cost and single point of failure, which is not cost-effective in the small-sized smart environment such as the smart home. One possible service management solution for WoT in smart homes is UPnP. UPnP is a Web-based industry standard that enables plug and play of devices in an IP-based home network. That is, as soon as a UPnP-compatible device is turned on, peer devices in the same home network are aware of its presence, capabilities, and the way of using its services. Technically, each UPnP device is a Web server that exposes a set of WS-* style Web Services [26,29]. The developers can create innovative services by programming a service manager that finds appropriate devices and then invokes the Web Services residing in the UPnP devices. The design of UPnP is optimized for smart homes, and its companion protocol, Simple Service Discovery Protocol (SSDP), is one of the few service discovery protocol that does not need a dedicated service directory.

**Figure 3:**
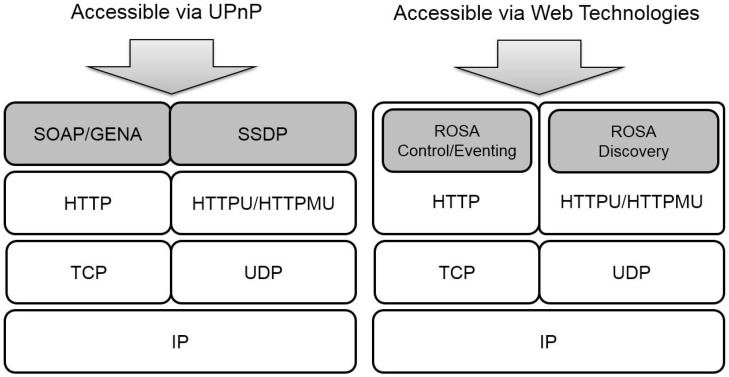
A comparison of protocol stacks between UPnP and ROSA.

However, as will be detailed in Section 3, the design of UPnP is not “web friendly” [10], making it hard to work with Web Things on the same home network. Thus, the purpose of this paper is, therefore, to design a set of Web-friendly service management schemes, called Resource-Oriented Service Administration (ROSA), for Web Things located in the same IP-based home network. The main contributions of this paper are (1) We formally specify the UPnP service management schemes, that is, discovery, description, controlling, and eventing of services. The formal notation is modified from [7,30] to make the notations fit in the context of this work; (2) We report the results of investigating the reasons that UPnP is not “Web-friendly” by examining the formal service management model of UPnP derived in the first step and evaluated it against a well-known and influential qualitative benchmark called RMM (Richardson Maturity Model) [9,31]; (3) We present ROSA, a set of Web-friendly service management schemes for smart homes. The protocol stacks of UPnP and ROSA are illustrated in Figure 3. It can be observed from this figure that UPnP defines a new protocol on top of HTTP/HTTPU(HTTP over UDP)/HTTPMU(HTTP over UDP Multicast) so that to interact with UPnP devices, a client must understand UPnP. On the other hand, ROSA’s philosophy is that instead of defining a new set of protocols, the functionalities of UPnP can be fully realized by HTTP/HTTPU/HTTPMU in regulated ways specified by ROSA schemes. Hence, the ROSA schemes allow the developers to access the sensors, software services, and home appliances through the standard Web technologies. The developers do not have to spend a lot of time learning and integrating all kinds of vendor-specific APIs or protocols.

To avoid neglecting others while considering one thing, UPnP is used as the baseline of the design of ROSA. In this way, ROSA is not only able to inherit design decisions from UPnP that make UPnP suitable for the small-sized home network but also being friendly to Web Things. For example, like UPnP, ROSA does not need a dedicated directory. To ensure that the resources can be accessed in uniform ways, this work also defines a resource model, denoted in Backus Normal Form (BNF), by following the design of UPnP Device Architecture. Finally, we implement a prototype of ROSA schemes and verify ROSA’s functional completeness, efficiency, and practical feasibility through experiments and implementation of the air conditioning service scenario mentioned above. We hope the results of this research will enable the developers with general Web technology backgrounds to control and set up the relevant Web Things on their own, and even go further to create new services.

## 2. Related Work

The system that coordinates and manages various devices and services in the smart environment is called a pervasive system in the research field of Pervasive Computing (PC) [32]. Pervasive systems are difficult to design and maintain because they involve heterogeneous hardware, software, wiring protocols, and programming paradigms. Typically, the pervasive system requires service management schemes to deal with the specific challenges mentioned above. Based on the architectural styles, pervasive service management schemes can be divided into two categories: centralized and decentralized. The centralized service management protocols collect and record all the status of managed nodes, namely the software services and hardware devices, to a dedicated directory server [33,34,35]. On the other hand, the decentralized service management schemes do not rely on a directory server, but to disseminate all the status of managed nodes based on multicast or P2P approaches, and then each node independently maintains the states of all nodes. SSDP/UPnP, APF(Artificial Potential Fields) [36] and SAPERE(Self-Aware Pervasive Service Ecosystems) [37] belong to this category. Zhu et al. [38] point out that, the decentralized service management schemes are more suitable for the small-sized smart environment such as a smart home because in such an environment, building up an additional directory server results in problems of high costs and single node failure. In a smart home, SSDP/UPnP seems to be an appropriate design because of its popularity and decentralized management architecture. However, UPnP possesses several problems and limitations itself. Thus, there have been many studies that focus on improving or expanding UPnP function. For instance, the research of Nakamura et al. [39] as well as Liao et al. [40] are committed to improving the effectiveness of the UPnP/SSDP service discovery mechanism. The research of [41] as well as [42] are mainly improving the efficiency of the UPnP event notification mechanism (GENA, General Event Notification Architecture); while Konstantinos et al. [43] strengthening the service performance and searching capacity of UPnP.

The concept of WoT first appeared in HP’s Cool Town project [44], which plans out a “Web-lized” world completely based on HTTP, each object possesses URL, which can be used to access the data or state of the object. Due to the limitation of the software technique at that time, the ideas failed to gain popularity. More recently, with the advances in computer hardware technologies and the raising of RESTful Web Services, many research works start to design pervasive systems by incorporating WoT concepts. For instance, Gurp et al. [1] considers the devices in the smart environment as Web resources, which can be accessed by any Web client. The others are to wrap up the sensor network based on WoT, enabling web clients to access data through RESTful API provided by the middleware [45,46]. These studies focus on device control and the access to data, without mentioning the issue of service management agreements. A group of researchers conducted a series of a systematic investigation on issues including platform, event notification, service management agreement and hardware feasibility when applying WoT in smart environments [2,5,13,47,48]. Their studies focus more on the sensor network of the large-sized outdoor space (the author calls it Sensor Area Network, SAN).

Based on 6LowPAN [49], even resource limited sensors and actuators connected by lossy and low power network (LLN) such as 802.15.4 or BLE can connect to the IP network transparently via an edge router. However, due to the different sizes of MTU(Maximum Transmission Unit) between traditional IP network and the LLN, existing popular application protocols for WoT in an IP network (i.e., HTTP and UPnP) can not work well in 6LowPAN. As a result, some works also suggest that a more compact way should be adopted to implement WoT and that HTTP should be replaced with binary coding in the large-sized WoT network, such as Constrained Application Protocol (CoAP) [22]. The functionalities of CoAP essentially mimics the design of HTTP and REST principles. However, as CoAP is UDP-based and the encoding scheme of CoAP is more compact than HTTP, CoAP is more suitable for accessing Web Things via 6LoWPAN. As a result, the application and integration of 6LoWPAN and CoAP in smart environments also become an active body of research [23,24]. In the past few years, service management issues for 6LoWPAN such as discovery [28,50,51], description using Semantic Web and Linked Data technologies [52,53], and controlling home appliances [54] have also been investigated. In [55], the authors present a complete framework for service management of Web Things in 6LoWPAN environments based on CoAP, RDF, and Linked Open Data (LOD) technologies. The authors also developed a web-based testbed for simulation and emulation with large-scale wireless sensor networks [56]. The testbed turns out to be very helpful for large-scale sensor network developers because their designs can be validated before the deployment of real sensors. As noted in [22], although 6LoWPAN makes nodes in LLN location-transparent to the IP network, two different application layer protocols with similar functionalities, namely HTTP and CoAP, are still needed for IP network and LLN, respectively. In similar rationale, different service management schemes need to be devised to work with HTTP and CoAP. As the objective of this work is to improve UPnP, we primarily focus on managing Web Things in IP-based home networks.

Pervasive REST Architectural Style (P-REST) expands and modifies REST according to the characteristic of the smart environment [57]. P-REST separates Uniform Resource Identifiers (URI) into two categories: Concrete URI (C-URI) and Abstract URI (A-URI). Among them, C-URI is the traditional URI, while the A-URI indicates that this URI is pointed to an abstract concept. The reason for separating different kinds of URI is because that group communication (e.g., IP multicast) is often required when realizing presence management and directory-less searching in a smart environment. An abstract URI serves as a virtual destination so that all nodes belonging to the same group be able to subscribe or send messages to this URI. This idea is similar to the use of virtual addresses from 224.0.0.0 to 239.255.255.255 for IPv4 multicast and ff00::/8 prefixed addresses in IPv6. The P-REST pattern offers additional HTTP Methods, include Access, Observe/Notify, and Lookup, which makes service management in the smart environment easier to be implemented. Unfortunately, this design brings about the same issue as the use of Notify and M-Search methods in UPnP: Existing web clients do not recognize these non-standard methods, resulting in the problem of compatibility. In this research, we take a different approach from P-REST and UPnP. To achieve better quality and high compatibility, the overall service management approach proposed in this paper, namely, ROSA, is carefully designed, so that it follows REST design guidelines and under the premise of not modifying the HTTP protocol. More concretely, ROSA is more promising than P-REST and UPnP in that ROSA provides the same set of service management functions without requiring additional HTTP Methods. The idea proposed in [58], which is the closest to this research, has improved the SOAP callback mechanism of UPnP with REST architecture, based on the concept of WoT. However, there is no further discussion about the UPnP’s service management issues, event notification, and other aspects.

A formal approach is used in this paper to present the service management schemes of UPnP and ROSA. Traditionally, a formal specification can be used in both statical (design time) and dynamic (runtime) ways. Ranganathan and Campbell [59] propose an approach that formally denotes the application logic of the Gaia platform using Ambient Calculus [60] so that the applications can be provably correct in the design time. On the other hand, in [61], the authors devise an approach for formal run-time verification in the context of service composition based on a CEP (Complex Event Processing) engine. Uri and Kedar’s work pioneered the research on formalizing and verification of RESTful services. In [30], they present a formal model and a set of notations for specifying RESTful HTTP-based interactions. Christian presents a UML-based domain model [62] and then adopts Uri and Kedar’s notations to specify IoT environments. Based on the specifications, the authors present an approach for automatically generating code segment of a Web Thing [63]. The IoT domain model present in [62] is similar to, but is also much simpler than, the UPnP Device Architecture. In this paper, the formal approach is used in design time. The notation presented in this paper is based on [30] with minor extensions and modifications to enhance the readability and to fit in the context of our work such as the need for group communications, multiple responses to a request, and A-URI [57].

## 3. Preliminary

As have been noted earlier, the primary objective is to design a service management scheme for WoT in a smart home. In this section, we will begin by briefly introducing the preliminary concepts of REST and an automated verifiable formal notation of RESTful behaviors, proposed by Uri and Kedar [30], which has been used to formally denote RESTful behaviors of a WoT system [62]. Next, we introduce the baseline model of our approach, namely UPnP Device Architecture and SSDP. On these grounds, we are then able to introduce our approach, ROSA, and explain how our approach supports discovering, describing, representing, and controlling of Web Things in the next few sections.

### 3.1. The REST Architecture and Its Maturity Model

The concept of REST was first described by Fielding in [7]. In REST architecture, all things are “resources” and all of the resources have a uniformed interface. The interface is defined by Methods of the HTTP standard [64], for example, GET, POST, PUT, DELETE, OPTIONS, HEAD, TRACE, CONNECT and PATCH. Each resources should be accessed by strictly following the semantics of these Methods. In this way, the resources are universally understandable and accessible by standard Web client following HTTP. From the perspective of object-oriented design, with the same interface, clients will not be affected with whatever implementation (a.k.a Polymorphism). This is also an important reason that we hope to implement the REST architecture in a smart home: regardless of the devices or the services underneath, as long as it meets the HTTP standard, the writing method of the clients in the smart home will be exactly the same as the general Web clients.

The above discussions drive us to an important issue: since the REST architecture is the key to implement WoT, does the REST architecture itself has a specific definition? How can it be considered as a RESTful design? How can the quality of a REST architecture design be verified? In fact, these questions are still in controversy. In other words, what is the design that meets the “REST standard” is still of a wide diversity of opinion, more research are required to clarify it [65]. So far, few works that claim itself to be “RESTful” examine and evaluate the quality of its design, at most, engage in initial experiments to show that REST architecture design has a higher efficiency than the WS-* type service. However, the breakthrough of REST and WoT must be focused on the improvement of the architecture, and that the efficiency is only a part of it as the advantages of REST architecture include: high scalability and efficiency, low coupling, interface uniformity and usability.

According to the literature that is known so far, RMM (Richardson Maturity Model) is one of the most influential indicators that is often used to evaluate the compliance of REST design principles. RMM was first proposed by Richardson in the keynote speech of QCon conference, and was further interpreted and commented by Fowler [9,31]. RMM has been proposed mainly because that the REST architecture does not have a clear specification definition as WS-* type Web Services, resulting in the uneven system quality of the REST architectural design. Hence, RMM is a qualitative model that could be used to assess the degree of REST design principles, based on the statement of REST in [7]. Concerning the validity of RMM, Fielding considers that only by reaching RMM Level 3 can be it be called the REST architecture (http://roy.gbiv.com/untangled/2008/rest-apis-must-be-hypertext-driven), while [31] argues that although the RMM model itself may not be sufficient to assess the quality of a RESTful service’s architecture, however, RMM is a very suitable tool for the continuous improvement of REST design. Thus, several recent studies have proposed metrics or benchmarks based on RMM for assessing the quality of RESTful systems [66,67].

In RMM, Level 0 is called the swamp of POX (Plain Old XML). As mentioned in the Introduction section, traditional SOAP-based Web Services belong to this type in the sense that HTTP is used as a transportation medium of remote invocations so that all invocation semantics were wrapped in the body of an HTTP message in the form of XML. Service endpoints use HTTP POSTs to trigger a remote invocation. Consequently, an RMM Level 0 service can not benefit from the design of REST architecture. RMM Level 1 is to model all service endpoints as resources on the Web, and RMM Level 2 takes it a step further that HTTP verbs are used as a universal service interface. This constraint effectively draws up a unified interface for the resources. In this way, clients can access all Web resources by following the same set of HTTP verbs, making the overall architecture loosely-coupled. RMM Level 3 refers to the resources that realize HATEOAS (Hypertext As The Engine Of Application State). More concretely, an RMM Level 3 service provides possible URIs that the client can access in its representation using hypermedia controls. The service typically follows ATOM (RFC4287) [68] standard and uses a “link” HTML element with an “uri” and a “rel” attribute to indicate the URI that the client can navigate.

In this work, we adopt RMM to guide us to improve the design of the service management scheme continually. Also, our design has been influenced by several guidelines on how to design a well REST services, such as the REST API design rules [69], the RESTful design patterns [70], and the Web API design instructions [8].

### 3.2. Formal Notations for RESTful Services

Formal methods are helpful for specifying the external observable behaviors (i.e., expected reactions to the specified requests) of RESTful services precisely and concisely. In [30,71], the authors proposed a theoretical basis that is applicable for using formal methods to model RESTful services, as well as carrying out the property derivation and verification. Reference [62] used these formal methods to specify the RESTful services deployed in smart spaces and IoT environments. As will shown in the next section, these studies are relevant references and formal tools for modeling, evaluation and design revisions in our research.

In this section, we introduce a set of formal notations for RESTful services used throughout this paper. The presented notations are modified based on [7,30] to make the notations fit in the context of our work. The noteworthy modifications are listed below:

- This work introduces a new semantic symbol “|” for denoting multiple responses. This enables one to specify group communications (e.g., HTTPMU) or to denote alternate behaviors (e.g., how the resources react to errors or the default behaviors for unexpected requests). Also, we introduce a new semantic symbol for A-URI [57] for group communication. A head (e.g., $\hat{a}$ ) added to a URI name denotes that it is an A-URI.
- The action concatenation symbol “/” is replaced by a bump equation “≎” as “/” appears frequently in URIs.
- We introduce a prefix for each action, *c* for Web client and σ for a root resource, to denote the performer of an action.
- We introduce the symbol of an empty set “∅”.
- Variable names are modified so that the expressions are easier to understand. For example, we use $A_{req}$, $A_{resp}$, and $A_{e}$ to denote the parameter sets of requests, responses, and errors, respectively.

The formal notations in this work are used in a block-box style [30] in the sense that it serves as a tool for specifying the expected reactions to certainly specified requests of UPnP and ROSA entities precisely. Therefore, we use the term “notation” instead of “model” or “specification” in this paper. In this way, the formal notations used in this paper primarily serves as a tool for conveying the design of schemes concisely and efficiently. Without the notation, the presentation of overall design can be vague and lengthy, because many fragments of HTTP request/response messages have to be shown to explain the idea. Besides, the introduction of formal notation in this paper also brings the following benefits: (1) The interactions among resources can be specified accurately so that the reproducibility can be enhanced; (2) The notations are helpful, in a language and platform independent way, for Web Thing developers when implementing ROSA schemes. Specifically, the formal notations serve as a guideline when implementing ROSA in Web Things. Since the formal notations provide a clear interface of expected inputs (what information is expected to receive) and outputs (what to respond when the request is processed successfully or when the specified node is not found); (3) The formal notation is helpful for making and presenting the arguments when the schemes are verified against RMM.

In a smart home, every accessible thing is called a resource and each resource is identified by one or more URI (Uniform Resource Identifier): each URI points to one resource whereas one resource can be pointed by different URIs. An accessible URI is called an URL (Uniform Resource Locator). Thus, to make a resource web-accessible, at least one of its URIs must be an URL so that a Web clients c∈C can access the resource via the URL. In this paper, we assume that all URIs are accessible and define a partial de-reference function η:I→R that maps URI i∈I to a resource r∈R.

Finally, Δ⊆I is the set of root URI identifying the root resource δ∈Δ that have unique IPs in a home network. Some device with powerful hardware may have embedded Web clients (called Control Points in UPnP). A resource has different representations (states) at a different time. Here a resource *r* is be represented by the set of states $\left\{d_{t_{1}},d_{t_{2}},\ldots d_{t_{n}}\right\}$ where *d* is the representation of *r* at time *t*. If we denote the universe of representations to be *D*, then a system of RESTful services can be formally represented using a tuple RS=(R,I,Δ,η,C,D). On these grounds, the behaviors in HTTP can be generalized as below:

(1) $$\vec{c}.m\left(i,A_{req}\right)≎\overset{\leftarrow }{\delta }.k_{resp}\left(d,A_{resp}\right)|k_{e}\left(d_{e},A_{e}\right)$$

The left-hand side is the request part, where a Web client *c* sends a request based on an HTTP method m∈M to a URI *i*. An optional set of parameters $A_{req}$ is sent with the request. In practice, $A_{req}$ can be either embedded in HTTP headers or as messages in the body. In the right-hand side, a device *b* receives the request and sends back a response message. The response message consists of a response code $k_{resp}$, current representation of *r*, namely *d*, and an optional set of response parameters $A_{resp}$. If the request is successfully processed, *b* uses a partial membership function υ:R→D to obtain the current representation *d* of *r*. Thus,

(2) $$d=\upsilon_{t_{current}}\left(\eta \left(i\right)\right).$$

Otherwise, *d* will be an error report carrying the description of the error. As shown in Expression (1), to make the specification concise, the default error processing behaviors (such as returning 500 Internal Server Error or 404 Not Found) are denoted uniformly using $k_{e}\left(d_{e},A_{e}\right)$, where $k_{e}$ denotes the return code, $d_{e}$ is the optional representation of the error and $A_{e}$ is an optional set of information encoded in the header of a message. However, the default error behaviors can also be overwritten when necessary.

In some cases, the parameter sets $A_{req}$, $A_{resp}$, or $A_{e}$ are empty sets. Then, these sets can be denoted as “∅” or, to make the expression concise, nothing at all. Namely,

$$\vec{c}.m\left(i,\emptyset \right)≎\overset{\leftarrow }{\delta }.k_{resp}\left(d,\emptyset \right)\left|k_{e}\left(d_{e},\emptyset \right)\equiv \vec{c}.m\left(i\right)≎\overset{\leftarrow }{\delta }.k_{resp}\left(d\right)\right|k_{e}\left(d_{e}\right).$$

However, the representations *d* and $d_{e}$ can not be skipped even when they are empty sets. Because in these cases, skipping “∅” causes ambiguities when identifying arguments between the parentheses.

Finally, if HTTP is used in a group communication (e.g., HTTPMU [72]), then a request can have multiple receivers, and thus leading to multiple responses. In these cases, the right-hand side of Expression (1) can be generalized as below to support multiple responses.

(3) $$\vec{c}.m\left(\hat{a},A_{req}\right)≎\overset{\leftarrow }{\delta }.k_{1}\left(d_{1},A_{resp1}\right)|k_{2}\left(d_{2},A_{resp2}\right)\left|\ldots \right|k_{n}\left(d_{n},A_{respn}\right),$$

where $\hat{a}$ is the abstract URI [57] for sending multicast requests.

## 4. Baseline Service Management Scheme

**Figure 4:**
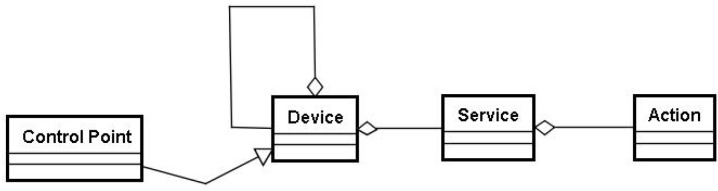
Service model of UPnP.

As mentioned, this work aims to propose a set of management scheme based on the service model of UPnP because of its popularity in home networks. Thus, before going into the details, we first briefly explain the service model of UPnP. The service model of UPnP is also known as the UPnP Device Architecture [26]. In this model, networked sensors, appliances, and software are called Devices. A Device contains one or more Services. Likely, a Service contains one or more Actions. Technically, an Action is a procedure that can be invoked remotely by following the SOAP protocol [73]. A Device that is capable of invoking Actions is called a Control Point. In this way, a UPnP network is made up of Devices, Services, Actions and Control Points. As shown in Figure 4, a Device may contain multiple sets of Services. A Service will contain multiple sets of Actions, and the Device itself can also contain multiple sub Devices and Control Points. In practice, an instance of a UPnP hardware usually has a Root Device. For example, a UPnP-enabled Indoor Light has a Light Root Device, and it includes a Dimming Service. The Dimming Service has one state field and two actions: Brightness State, Increase Brightness Action and Decrease Brightness Action. Based on UPnP Device Architecture, we are ready for presenting the service management scheme of UPnP. The UPnP specification divides service management scheme issues into four aspects and specifies them in the Discovery, Control, Eventing, and Description sections, respectively [26].

### 4.1. Discovery

The Discovery mechanism is carried out by SSDP, which consists presence announcement (PA), leave announcement (LA), and proactive search mechanisms. By default, SSDP operates based on HTTPMU (HTTP over UDP multicast). HTTPMU uses IP multicast, which forwards packets to a group of interested receivers. In this way, SSDP does not need a centralized service registry since IP multicast is carried out by the underlying infrastructure (i.e., the network switch). SSDP extends HTTP with two methods: NOTIFY and M-SEARCH. The NOTIFY method is used for announcing presence and absence of UPnP devices, namely PA and LA; M-SEARCH is used for searching UPnP devices of a specific type or URI, namely the proactive search. As a result, SSDP consists of three primitive actions: (1) ssdp:alive: announces the presence of a node; (2) ssdp:byebye: announces that a node has left the network; and (3) ssdp:discover: attempts to find a node that meets the type specified in ST (Search Target) header in an M-SEARCH message. The matched devices then reply by an HTTP Response message.

**Figure 5:**
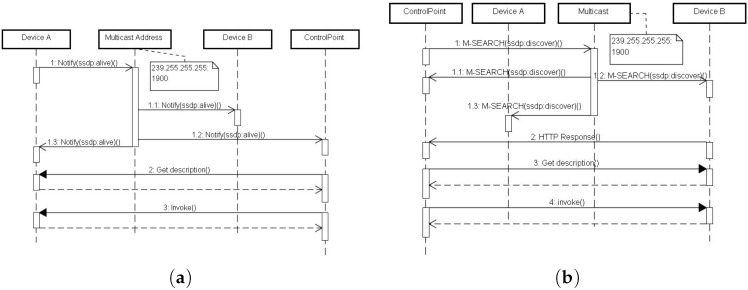
Baseline service management scheme: (**a**) UPnP presence and absence announcement; (**b**) UPnP proactive search.

Whenever an UPnP Device is started/stopped, it sends PA/LA messages to the multicast address to inform other UPnP Devices about its presence/absence. Formally,

(4) $$\vec{\delta }.NOTIFY\left(\hat{a},\left\{ssdp:alive,i_{\delta },i_{\delta }^{desc},\tau_{\delta }\right\}\right)≎⊥$$

for PA, and

(5) $$\vec{\delta }.NOTIFY\left(\hat{a},\left\{ssdp:byebye,i_{\delta }\right\}\right)≎⊥$$

for LA, where δ is the device, $\hat{a}$ is the multicast address, $i_{\delta }$ is the URI of δ, $i_{\delta }^{desc}$ is the URI of description document, $\tau_{\delta }$ is the type of δ, and ⊥ denotes that no response is expected. The PA/LA messages are then propagated to all other UPnP Devices. As shown in Figure 5a, both devices and Control Points can receive a NOTIFY of ssdp:alive from Device A. For example, in Figure 5a, Control Points and Devices receive PAs and use this information to maintain the local copy of the “view” (A collection of identities of the UPnP Devices that are alive) of the local network.

Alternatively, a Control Point can also search for UPnP Devices with a specific type. As mentioned, in SSDP, this is done by issuing an M-SEARCH request to the multicast address. Given that a Control Point *c* wants to search for UPnP Devices with type τ, it first sends an M-SEARCH to the multicast address $\hat{a}$, and then *c* receives a response message if there is a match, otherwise, there is no response (see Figure 5b). Formally,

(6) $$\vec{c}.MSEARCH\left(\hat{a},\left\{\tau \right\}\right)≎\overset{\leftarrow }{\delta }.200OK\left(\emptyset ,\left\{i_{\delta },i_{\delta }^{desc},\tau_{\delta }\right\}\right)|⊥,$$

where δ is the device such that $\tau =\tau_{\delta }$ and “∅” denotes that no representation is returned.

### 4.2. Description

By using the Discovery mechanism, a Control Point can search for a device via either ssdp:alive or ssdp:discover. Yet the Control Point still has limited information about the device (URI and the device’s type). Thus, a description document is required to find out more details before the Services and Actions of a Device can be invoked. UPnP uses a customized XML-based representation format to reveal Devices, Services, Actions contained in a Root Device. For example, as shown in the Step 2 in Figure 5a and Step 3 in Figure 5b, a Control Point gets the description document from the URIs ($i_{c}$ in Expression (4) or $i_{b}$ in Expression (6)) contained in the PA or M-SEARCH messages, formally,

(7) $$\vec{c}.GET\left(i_{\delta }^{desc}\right)≎\overset{\leftarrow }{\delta }.200OK\left(d_{\delta }^{desc}\right),$$

where *c* is the Control Point, $i_{\delta }^{desc}$ is the URI of description document, δ is the discovered device, and $d_{\delta }^{desc}$ is the description document of δ.

### 4.3. Control

After examining the contents of description document, the Control Point knows what Services and Actions can be invoked and how to invoke them, as shown in Step 3 of Figure 5a and Step 4 of Figure 5b. In UPnP, the invocation mechanism is a customized version of SOAP [73]. Technically, the Control Point invokes a remote Action by sending a POST request following the URI of the Action, where the URI can be obtained by looking up the description document. The invoked Action either returns the execution results or error messages wrapped by a SOAP envelope. Formally, is a match, otherwise, there is no response (see Figure 5b). Formally,

(8) $$\vec{c}.POST\left(i_{\left(\delta ,a\right)},d_{invoke}\right)≎\overset{\leftarrow }{\delta }.200OK\left(d_{results}\right)|k_{e}\left(d_{e}\right),$$

where δ is the device containing the Action *a* to be invoked, $i_{\left(\delta ,a\right)}$ is the URI of the device, $d_{invocke}$ is the invocation context wrapped using a SOAP envelope, $d_{results}$ is the returned SOAP envelope containing results, $k_{e}$ denotes the HTTP error status code (typically 400-899), and $d_{faults}$ is the error messages wrapped by a SOAP envelope.

### 4.4. Eventing

UPnP also defines an eventing mechanism called GENA (General Event Notification Architecture) so that a Control Point can react to stage changes of devices. General speaking, GENA works in a way similar to the Observer design pattern [74]. The Observer and the Subject are main roles in this pattern, where the Subject notifies the Observer if the value of interested states changes or when some interesting events happen. In GENA, two additional HTTP methods are added: SUBSCRIBE and UNSUBSCRIBE to indicate subscription and un-subscription of state changes. Besides, the NOTIFY method (originally used for PA/LA) is employed in GENA for event notification.

To observe a state change event of a UPnP Service, the Control Point (Observer) first registers interested states by sending a SUBSCRIBE request to the URI of the Service (Subject). Based on the UPnP specification, the URI can be found in the “eventSubURL” element of the description document. Also, the Control Point must specify a URI that is to be called by the Service when performing notification and the desired subscription period. After accepting the subscription, the Service returns an HTTP response containing an ID for tracking this subscription and a timeout indicating the approved valid period of this subscription. Otherwise, an error code is returned (typically 400, 412 or 5xx). The overall subscription procedure in GENA can be summarized below:

(9) $$\vec{c}.SUBSCRIBE\left(i_{\left(\delta ,s\right)},\left\{i_{callback},t_{requested}\right\}\right)≎\overset{\leftarrow }{\delta }.200OK\left(\emptyset ,\left\{\iota ,t_{approved}\right\}\right)|k_{e}\left(d_{e}\right),$$

where $i_{\left(\delta ,s\right)}$ is the URI of the Service *s* in the device δ to be subscribed, $i_{callback}$ is the callback URI, $t_{requested}$ is the requested subscription period, ι is the subscribed URI, $t_{approved}$ is the approved subscription period, $k_{e}$ is the error code, and $d_{e}$ is the error description. Then, once the observed state is changed, the Service that contains the changed state sends notification to all subscribers by issuing a NOTIFY request to all registered callback URIs ($i_{callback}$):

(10) $$\forall i\in I_{callback},\vec{\delta_{s}}.NOTIFY\left(i,\left\{P_{s}\right\}\right)≎\overset{\leftarrow }{c_{i}}.200OK\left(\emptyset \right)|k_{e}\left(d_{e}\right),$$

where $I_{callback}$ is the set of subscriber’s callback URIs, $\delta_{s}$ is the device containing the subscribed Service *s*, $P_{s}$ is the set of key-value pairs describing the names and values of changed states, $c_{i}$ denotes the subscribing Control Point identified by the URI *i*, $k_{e}$ is the error code, and $d_{e}$ is the error description. Similar procedure applies to un-subscribing a Service and is not repeated here.

### 4.5. Discussion

Apparently, the design of UPnP’s RMM is Level 0. By examining Expressions (4)–(10), we have identified the key factors that cause UPnP to be RMM Level 0, which are summarized below.

- Proprietary way of client state transfer: After a Control Point (the client) finds a UPnP Device, the next step is to retrieve the description document revealing the services that can be invoked. To be precise, to follow the HATEOAS convention the URI *i* of the description document in Expression (7) should be obtained using hypermedia controls and thus following either following the Web Linking (RFC 5988) [75] or the ATOM (RFC 4287) to ensure interpretability. Unfortunately, UPnP does not follow the HATEOAS convention and only offers a “fake hypermedia” [8] (i.e., a proprietary way of URI provision), leading to the disqualification of RMM Level 3 (Hypermedia Controls).
- Proprietary extension and the overloading of HTTP verbs: UPnP uses a lot of non-standard HTTP verbs for supporting discovery (NOTIFY and M-SEARCH, see Expressions (4)–(6)) and eventing (SUBSCRIBE, UNSUBSCRIBE, and NOTIFY, see Expressions (9) and (10)). On the contrary, as can be observed in Expression (8), UPnP makes use of an overloaded POST for all remote invocations without considering the semantics of these calls. Also note that in the design of UPnP, there is some illegal use of HTTP response code. For example, in Expressions (6), (9) and (10)), the responses do not contain any body and thus they should return 204 No Content instead of 200 OK. The issues mentioned above cause the disqualification of RMM Level 2 (HTTP Verbs).
- Singular service endpoint: In UPnP, there is one endpoint for each service, in other words, no matter how many embedded UPnP Actions does a service provides, the only way to access these Actions is through the URI. To call an Action, the invocation contexts are wrapped in the body of a POST request to the URI (see Expression (8)). As a result, a UPnP device is not designed as a Web Resource and thus prevent UPnP from conforming to RMM Level 1 (Resources).

Judging from above, it is fair to say that despite the popularity and comprehensiveness of UPnP, the service model of UPnP, which is RMM Level 0, does not conform to the design philosophy of the Web and thus is not suitable for emerging WoT-based smart homes.

## 5. Resource-Oriented Service Administration

This section describes the design of a Web-friendly (RMM Level 3) service management scheme based on UPnP called ROSA (Resource-Oriented Service Administration). As suggested by [31], the first step toward the RESTful architecture is to break a singular service endpoint into Web resources (called resources in the sequel). To make it clear, let us take the Indoor Light Root Device mentioned in Section 4 as an example and assuming that LIGHT-IP is the IP address of the device. To remotely obtain the value of brightness level of the Indoor Light Root Device, the Control Point send a POST request to the device. In the POST request, the invocation context including the Action name and values of arguments are wrapped in a SOAP envelope, as shown below (to make the XML more readable, the namespace information is removed):

POST /_urn:my-domain:serviceId:DimmingService HTTP/1.1
Host: LIGHT-IP
<envelope>
  <body>
    <GetBrightnessLevel><RetBrightnessLevelStatus/></GetBrightnessLevel>
  <body>
<envelope>

In the REST way, everything is a resource and is identifiable and accessible via a URI. Thus, each Device, Service, and Action has an unique URI *i* such that the representations can be obtain by υ(η(i)) (see Expression (2)). Thus, we define a URI naming syntax (see Listing 1) for all UPnP entities by extending the generic syntax of RFC 2396 [76] so that each UPnP entity has a unique URI and thus is accessible by Control Points (called Web clients in the sequel) in a smart home. In the UPnP-enabled Indoor Light example, then the Web client can get the brightness value of the light by issuing an HTTP GET request to the resource URI:

GET /LightDevice/DimmingService/Brightness HTTP/1.1
Host: LIGHT_IP.

The point to observe is that to construct a RESTful service, the domain model of UPnP (Device, Services, Services’ states, and Actions and their hierarchical relationships) is making explicit as resources where the resources can be accessed universally by Web clients using HTTP verbs. Hence, the service is designed so that HTTP is its “architectural style.” On the contrary, in the UPnP approach, the invocation context is wrapped in the HTTP message body so that HTTP is its "transportation medium”.

Listing 1: The BNF of URIs of ROSA resources, PCHAR denotes printable characters, SERVER is the Server-based Naming Authority of the form <userinfo>@<host>:<port>falselst:ROSAURI
ROSA_URI = ’http://’ SERVER ROOT_DEVICE [EBD_DEVICE]
                     SERVICE STAT_VAR [ ’?’ QUERY_PARAMS ]
ROOT_DEVICE = ’/’ *PCHAR
EBD_DEVICE = ’/’ *PCHAR  *[’/’ *PCHAR ]
SERVICE = ’/’ *PCHAR
STAT_VAR = ’/’ *PCHAR
QUERY_PARAMS = *PCHAR ’=’ *PCHAR

The next step is to follow the design structure for a service management scheme proposed in UPnP and by Stirbu [47]. In the following subsections, we present the WoT-enabled service management scheme from the following aspects: Discovering and describing things, Controlling things, and Notifications from things.

### 5.1. Discovering and Describing Things

Recall that PA, LA, and proactive search make up the Discovery mechanism. It can be observed from Expressions (4) and (5) that to implements PA and LA, UPnP introduces a proprietary extension of HTTP verb (NOTIFY). In addition, an additional NTS header is required to distinguish PA from LA (an ssdp:alive is for PA and an ssdp:byebye is for LA). The main reason for this extension is that the communication style of Discovery mechanism, typically group communication (one to many or many to one), is different from the communication style of HTTP (request-response and one to one). However, as mentioned, this design lead to disqualification of RMM Level 2.

**Figure 6:**
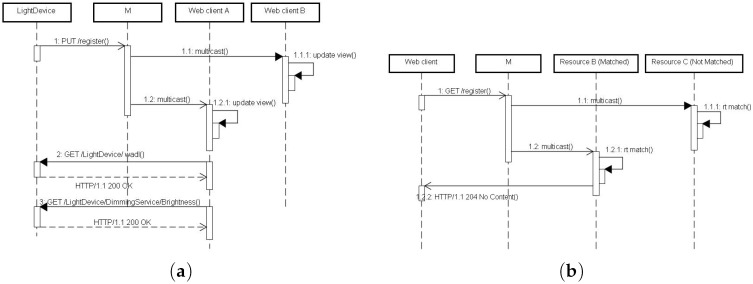
Proposed service management scheme: (**a**) ROSA presence announcement; (**b**) ROSA proactive search

Our approach is inspired by the A-URI (Abstract URI) concept [57]. The multicast address $\hat{a}=$ 239.255.255.250:1900 is seen as a virtual resource which is identified by an A-URI and plays the role of a virtual publicly available centralized registry for all other resources. In this way, it becomes intuitive that the semantic of sending a PUT to the registry is to perform PA and sending a DELETE to the registry is to perform LA. Let us first define an A-URI (http://239.255.255.250:1900/registry) of a virtual resource which serves as the registry for all other resources in the smart home. To perform a presence announcement, a WoT-enabled television device PUTs the following message to the A-URI (see Figure 6a, Step 1):

PUT /registry HTTP/1.1
Host: 239.255.255.250:1900
URI: (URI of the device)
RT: <http://percomlab.org/rosa/rt/TV>
Link: <http://192.168.4.101:60001/wadl>;rel="http://percomlab.org/rosa/api"

where RT is a predefined resource type (TV), and the LINK header is the URI to its description document, following the Web Linking standard (RFC 5988) [75]. Note that the URI (http://192.168.4.101:60001/wadl) implies that format of the description document is WADL [77]. Our approach allows the provision of other formats of description document such as WTM (Web Thing Model) [15], OAS (OpenAPI Specification) (https://www.openapis.org/) or RAML (RESTful API Modeling Language) (https://raml.org/) by using different tailing URIs /oas and /raml, respectively. Also note that, following the suggestion of RFC 5988, “rel = http://percomlab.org/rosa/api” is used to refer an extension relation type of Web API description document. Formally,

(11) $$\vec{\delta }.PUT(\hat{a}/registry,\left\{\left\{i_{\delta },i_{\delta }^{desc},\tau_{\delta }\right\}\right)≎⊥$$

The benefits of this approach are (1) by introducing the concept of A-URI, the multicast address $\hat{a}$ is seen as an identifiable and accessible Web resource; (2) no proprietary extension of HTTP verb is required and (3) no additional (NTS) header is required.

As $\hat{a}$ is a multicast address, the PUT request message is then received by all peer resources in the network (see Figure 6a, Steps 1.1 and 1.2). Then, an interesting Web client can get the Web API description document by following the URI provided in the LINK header (see Figure 6a, Step 2):

(12) $$\vec{c}.GET\left(i_{\delta }^{desc}\right)≎\overset{\leftarrow }{\delta }.200OK\left(d_{\delta }^{desc}\right).$$

Similarly, a leaving resource can send a DELETE request to the A-URI to notice its absence:

(13) $$\vec{\delta }.DELETE\left(\hat{a}/registry,\left\{i_{\delta }\right\}\right)≎⊥.$$

Following the concept of A-URI, to search for a specific type of resource, the Web client first sends a GET to the virtual registry requesting the resources with a specific type (see Figure 6b, Step 1). If there is a match, the matching resource (δ) will reply by send an HTTP response indicating its URI ($i_{\delta }$) and the URI of description document ($i_{\delta }^{desc}$) for further access, otherwise there is no response (see Figure 6b). Note that the return code is 204 as there is no body part in the response message:

(14) $$\vec{c}.GET\left(\hat{a}/registry,\left\{\tau \right\}\right)≎\overset{\leftarrow }{\delta }.204NoContent\left(\emptyset ,\left\{i_{\delta },i_{\delta }^{desc}\right\}\right)|⊥.$$

### 5.2. Controlling Things

Based on the purposes, one can consider Control in UPnP under two types: (1) to query the status of a service and (2) to change the state of a service. As mentioned, in UPnP, both types are realized by an overloaded POST request which is wrapped by a SOAP envelope. In ROSA, everything in the smart home is seen as a resource, and each of the resources can be identified by a URI (see Listing 1). From what has been discussed above and by considering the protocol semantics of HTTP verbs, it is evident that the RESTful way of querying the status of a service is to send GET requests to the URI of the service status variable. Formally,

(15) $$\vec{c}.GET\left(i_{\left(s,v\right)}\right)≎\overset{\leftarrow }{\delta }.200OK\left(d_{results}\right)|k_{e}\left(d_{e}\right),$$

where *c* is the Web client, *s* is the service containing the status variable *v* to be queried, $i_{\left(s,v\right)}$ is the URI of the variable, $d_{results}$ is the returned representation containing the status, $k_{e}$ denotes the HTTP error status code, and $d_{e}$ is the error messages. Note that the proposed scheme does not set any constraint on the document type of $d_{results}$ and $d_{e}$. Except for the SOAP envelope, ROSA reuses the document type in UPnP. One concrete example has been provided in the Indoor Light Root Device example at the beginning of Section 5, where a GET is issued to the URI of the Brightness status variable.

The second type of Control is to change the state of a service. In the Indoor Light Root Device example, invoking the Increase Brightness Action causes a update (increasing) of brightness level. As a result, PUT semantically fits this purpose, that is,

(16) $$\vec{c}.PUT\left(i_{\left(s,v\right)},d_{new}\right)≎\overset{\leftarrow }{\delta }.204NoContent\left(\emptyset \right)|k_{e}\left(d_{e}\right),$$

where *c* is the Web client, *s* is the service containing the status variable *v* to be updated, $i_{\left(s,v\right)}$ is the URI of the variable, $k_{e}$ denotes the HTTP error status code, and $d_{e}$ is the error messages. Note that if the state update is successful, then the message body is not needed in the the response, that is, 204 No Content should be used as the response code instead of 200 OK.

### 5.3. Notification from Things

Similar to the design of GENA, if a Web client *c* wants to observe the state changes of a service *s* in a root device δ, *c* first subscribes to such events by sending a PUT to the URI, denoted $i_{\left(\delta ,s\right)}^{v}$, of the state variable *v*. Meanwhile, the URI of the Web client $i_{c}$ and the subscription period $t_{requested}$ is also sent as parameters. Again, after accepting the subscription, an HTTP response containing an ID, denoted ι, for tracking this subscription and a timeout $t_{approved}$ indicating the approved valid period of this subscription are returned; Otherwise, an error code $k_{e}$ is returned with a document $d_{e}$ that explains the error. As a result, the overall subscription procedure in ROSA can be summarized below:

(17) $$\vec{c}.PUT\left(i_{\left(\delta ,s\right)}^{v},\left\{i_{c},t_{requested}\right\}\right)≎\overset{\leftarrow }{\delta }.200OK\left(\emptyset ,\left\{\iota ,t_{approved}\right\}\right)|k_{e}\left(d_{e}\right).$$

The main difference with GENA subscription is that no additional HTTP method (SUBSCRIBE) is required. Instead, the canonical HTTP verb PUT is used to indicate “an update to the subscriber list of the state.” A similar procedure applies to un-subscribing a state of a resource and is not repeated here.

When the subscribed state is changed, notifications are sent to all subscribers by issuing a PUT request to all registered callback URIs ($I_{callback}$):

(18) $$\forall i\in I_{callback},\vec{\delta_{s}}.PUT\left(i,\left\{P_{s}\right\}\right)≎\overset{\leftarrow }{c_{i}}.200OK\left(\emptyset \right)|k_{e}\left(d_{e}\right),$$

where $I_{callback}$ is the set of subscriber’s callback URIs, $\delta_{s}$ is the device containing the subscribed Service *s*, $P_{s}$ is the set of key-value pairs describing the names and values of changed states, $c_{i}$ denotes the *i*th subscribing client identified by the URI *i*, $k_{e}$ and $d_{e}$ are respectively the error code and the error description. Likely, the main difference with GENA notification is that no additional HTTP method (NOTIFY) is required. Instead, PUT is used to indicate “an update to the client copy of the subscribed state.”

## 6. Evaluation

In this section, we present the results of evaluating Web Friendliness, feasibility, and performance of ROSA. The details are presented in the next few sections.

### 6.1. Web Friendliness

Web Friendliness is defined as “the degree that meets the REST design principles.” [10]. A service with high RMM level indicates the high Web Friendliness, and thus the service has the benefits of high scalability, high efficiency, low coupling, interface consistency, and usability, as promised by REST architecture. In this subsection, we assess the Web Friendliness of ROSA based on RMM from perspectives of discovering and describing things, controlling things, and notification from things.

- Discovering and describing things: Main tasks of discovering and describing things include PA, LA, proactive search, and accessing description document. Expressions (11)–(14) respectively specify the behaviors of PA, LA, proactive search, and the access of description document in ROSA. By introducing the concept of A-URI, the multicast address $\hat{a}$ is seen as a resource, making the design conform to RMM Level 1. Furthermore, the design conforms to Level 2 as all non-standard HTTP verbs such as NOTIFY and M-SEARCH are replaced by canonical HTTP verbs (PUT and GET) (see Expressions (11), (13) and (14). Finally, both the request messages (Expression (14)) provide Web Link to the description document of the resources so that they reveal HATEOAS semantics. In other words, in ROSA, PA/LA and proactive search are RMM Level 3. In the case of description, the conformance to RMM Level 3 depends on the description format. For example, if WTM is used, then describing things in ROSA is RMM Level 3: in WTM, there is a “link” attribute for navigating to another resource.
- Controlling things: Key tasks of controlling things include getting the status of a service variable and change the state of the variable which is respectively specified in Expressions (15) and (16). It is obvious to see that, in ROSA, the schemes of controlling things conform to RMM Level 1 as the variable is also exposed as resources and can be accessed via URIs (i.e., $i_{\left(s,v\right)}$ in Expressions (15) and (16)). The design also conforms to Level 2 as overloaded POSTs are replaced by canonical HTTP verbs (PUT for changing the states and GET for querying the states). Note that there is no HATEOAS semantics for controlling things as the subsequent control commands should be sent to the same URI.
- Notification from things: Notification in ROSA includes subscribing a specific state change event of a service variable and publishing state change events to the subscribers. According to Expressions (17) and (18), a service variable that can be observed and the set of targets of notification are modeled as resources, respectively $i_{\left(\delta ,s\right)}^{v}$ and $I_{callback}$. Thus, notification in ROSA conforms to RMM level 1. Furthermore, the design also conforms to RMM level 2 as there is only one canonical HTTP verb PUT used in our design: for both modifying the observer list of a service variable and notifying the state change events. Subscribing a state change in ROSA is RMM level 3 because a URI, denoted by *i* in the response part of Expression (17), is provided for tracking the subscription. Again, the notification part is not RMM level 3 as there is no HATEOAS semantics for event notification in the baseline scheme (i.e., GENA).

To sum up, all schemes in ROSA are at least RMM Level 2, and most of them are Level 3. The exceptions include the description (depends on the description format), control and change notification (no HATEOAS semantics required according to the baseline schemes. To make all ROSA schemes conform to RMM Level 3, an HATEOAS-capable description format should be enforced, and links to additional information have to be added in the headers of response messages of control and event notification. However, the price to pay is the flexibility (fixed description format) and complexity (possibly unnecessary features). Thus, it is fair to say that ROSA is a “Web Friendly” service management mechanism as most of its schemes have high RMM levels.

### 6.2. Application Prototype

We verify the feasibility of ROSA by realizing a proof-of-concept prototype air conditioning service in a smart home illustrated in Figure 2. This prototype consists of an IP network, a ZigBee-based wireless sensor network (configured as simple star topology), and one proprietary wireless home automation network made by Techcity (http://e2-live.com/techcitysite/). The ZigBee-based sensor network consists of two end devices, denoted as Temperature Sensor Node 1 & 2, and one coordinator denoted as WSN Coordinator. The end devices are made using two Arduino Uno boards with XBee/S2 (set as ZigBee Router AT mode) and temperature sensors attached. The WSN coordinator is made using a Raspberry Pi 3 with an Arduino Uno and a XBee/S2 (set as ZigBee Coordinator API mode) attached. In the Raspberry Pi 3, we also wrote and deployed a program, implemented as a Web Thing (WT1), that collects the signals from the sensor network and exposes the sensed values based on ROSA. The home automation network consists of a Control Gateway (Techcity HC-12), which receives UDP-based vendor-specific commands and then control the air conditioners accordingly via infrared signals. To bridge HC-12 and IP network, we implemented and deployed a Web Thing (WT5) for controlling the air conditioner. WT5 receives JSON-based control commands conforming to the ROSA schemes, translates the commands, and sends the translated command using UDP to HC-12. The Web Thing WT2 is implemented using Arduino Uno WiFi with a temperature sensor attached. The firmware of WT2 is programmed so that it exposes the sensed values as a resource and can be accessed by following ROSA schemes. WT3 and WT4, deployed on a PC, are ROSA-based Web Things that realizes the context interpretation and the action reasoning logics, respectively. Among the Web Things, WT1, WT3, WT4 are implemented using JavaScript on top of Node.js [78]; WT2 is realized using Arduino sketch; WT5 is implemented using Java (JDK 8) and the Apache CXF library (an implementation of JAX-RS specification) [79]. To observe the communication messages of ROSA, we also setup a laptop with the Wireshark packet analyzer (https://www.wireshark.org/) installed so that so that it is easier to ensure that the results are correct.

Like UPnP, the Web Things managed by ROSA are located in the same local area network (LAN) so that they can be discovered through IP multicast. The discovery mechanism of ROSA operates based on IP multicast. WT5 makes use of the *HTTPMUSocket* class provided by the Cybergarage UPnP framework (https://github.com/geniusgithub/dlna_framework). Meanwhile, the discovery logics of WT1, WT3, and WT4 are constructed using the *dgram* library provided by Node.js. For WT2 (Arduino Uno WiFi), the multicast is realized using the WiFiUdp library. In this prototype system, the description of Web Things is written in JSON format following the WTM specification [15]. The controlling things and notification from things behaviors are built on top of HTTP libraries provided by Apache CXF (Java) and Express.js [80] for WT0 and WT1, 3, 4, respectively.

The following example shows how a developer implements the TemperatureService located at WT1/Node1 by referring to Expression (15) using JavaScript, the JohnnyFive API and the Express.js web framework. Expression (15) indicates that the service reacts to a GET request. Also, in the request, the name of the service *s* and the queried variable *v* are passed as parameters (reflected in line 5 of Listing 2). Then, the response part (right-hand side) of Expression (15) reveals that the service either returns 200 with a result message (lines 10–13) or returns an error code and a message (lines 17–19). As the formal notations provide a clear interface of expected inputs (what information is expected to receive) and outputs (what to respond when the request is processed successfully or when the specified node is not found), these notations turn out to be useful for guiding a developer when implementing the Web Thing.

Listing 2: Partial implementation of temperature service sensor nodes managed by WT1falselst:impl:GET1

1 
2 let measurements = ..// a map of latest measurements of nodes
3 let nodes = ...// get a list of names in the ZigBee network periodically
4 
5 router.get(’:node/TemperatureService/measurement’, function (req, res, next) {
6 
7 
8     nodes.forEach((node)=>{
9       if(node === req.params.node) {
10           let responseMessage = {};
11           responseMessage.temperature = measurements [node];
12           res.status (200).json (responseMessage);
13           next();
14         }
15     });
16 
17     responseMessage.description = "No Such Node.";
18     res.status(404). json (responseMessage);
19     next();
20 });
21 ...

We are now able to demonstrate how ROSA works in the air conditioning service scenario. As mentioned, a service is composed and maintained by a dedicated service manager. A service manager is driven by a service specification, which specifies the functional types of the desired Web Things. In this scenario, the air conditioning service, requires two Web Things that provide temperature information, two Web Things of the functional types Context Interpreter and Action Reasoner, respectively, and one Web Thing of the Air Conditioner functional type. When the composition process begins, the service manager first searches for all Web Things with the functional types mentioned above by performing the proactive search (see Figure 6b) scheme specified in the left-hand side of Expression (14):

$$\vec{c}.GET\left(239.255.255.250:1900/registry,"temperatureSensor"\right).$$

**Figure 7:**
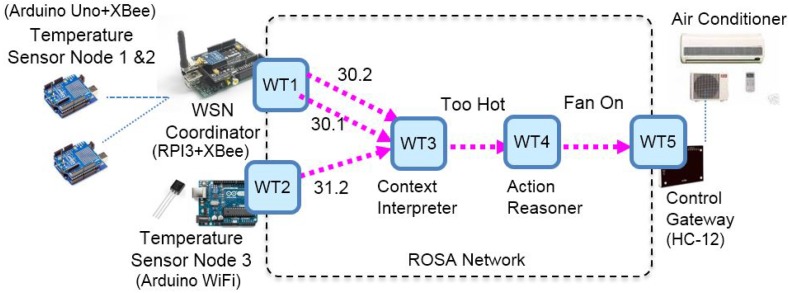
The prototyped ROSA application scenario; The dotted lines indicate dataflow.

Note that 239.255.255.250 is the multicast address assigned to UPnP. The address should be replaced by FF00::C, IPv6 Multicast Address Space Registry, available on line at (https://www.iana.org/assignments/ipv6-multicast-addresses/ipv6-multicast-addresses.xhtml) if IPv6 is used. For clarity, we will use IPv4 in the sequel. After receiving the search message, WT1, for instance, returns the following response message because its functional type matches “temperatureSensor”:

$$\overset{\leftarrow }{\delta_{WT1}}.204NoContent\left(\emptyset ,\left\{192.168.4.21/WT1,192.168.4.21/WT1/wtm\right\}\right),$$

where 192.168.4.21/WT1 is the URL of WT1 root resource and 192.168.4.21/WT1/wtm is the URL of its WTM description file. Other Web Things can be discovered in similar ways. The service composition is successful if and only if all needed Web Things with required functional types are found. After the service is composed, the service manager has to periodically invokes the TemperatureService of WT1 to obtain current temperature measurements. The URL of invocation can be obtained by examining WTM of WT1. There are two sensors nodes, Node1 and Node2, in the same sensor network managed by WT1 (see Figure 7). The measurements of these nodes can be accessed respectively via 192.168.4.21/WT1/Node1/temperatureService and 192.168.4.21/WT1/Node2/temperatureService. After getting temperature measurements, the service manager then obtains the desire actions through invoking the services of WT3 (Context Interpreter) and WT4 (Action Reasoner). This can be implemented by Expression (15). Below is a sequence of possible requests and responses:

$$\vec{c}.GET\left(192.168.4.23/WT3/contextInterpreterService,\left[30.2,30.1,31.2\right]\right)$$

$$≎\overset{\leftarrow }{\delta_{WT3}}.200OK\left(\left\{situation:TooHot\right\}\right)$$

and

$$\vec{c}.GET\left(192.168.4.23/WT4/actionReasonerService,\left[TooHot\right]\right)≎\overset{\leftarrow }{\delta_{WT4}}.200OK\left(\left\{command:FAN\_ON\right\}\right).$$

Now, as WT4 suggests the service manager issue a FAN_ON command to WT5. This can be done by invoking airconditioningService in WT5 with a PUT (c.f. Expression (16)), which translates the command and turn on the fan of the air conditioner:

$$\vec{c}.PUT\left(192.168.4.24/WT5/airconditioningService/fan,ON\right).$$

### 6.3. Experimental Section

The overall experiments can be divided into two parts. The first part of experiments tests the performance of service discovery using ROSA. Specifically, the objective is to study how the elapsed discovery time is affected by the number of existing Web Things and the size of services (the number of things to find in a round).

When the experiment begins, the service manager (a Web client) performs a ROSA search (Expression (14)) by sending an HTTP request via HTTPMU. The target resource types are specified in the search messages. Then, the service manager receives responses from the matching Web Things. Theoretically, given that there are *m* managed Web Things and totally *n* distinct resource types, if the resource types are uniformly distributed, then the service manager receives γ·m/n responses for every search, where γ is the number of distinct resource types to be discovered. There is a trade-off between the quality and efficiency of search results. To enhance the efficiency of searching, the service manager stops searching when it receives the first matching response (i.e., the first come, first selected policy). On the other hand, to emphasize the search quality, the service manager must wait until all matched messages are received so that the best matching candidate can be selected. In this experiment, we evaluate and compare both quality-first and efficiency-first policies. All Web Things are implemented using Java and Apache CXF. The Web Things are evenly distributed over three Intel Core i5 CPU with 4G memory PCs in the same LAN. All PCs connected to a 150M bps router in the experiments. The service manager is programmed to search for three Web Things with three specific resource types (γ=3). All Web Things emit PA messages every 5 s (Expression (11)). In the experiment, resource types are randomly assigned. The assignment must meet the constraint m/n≥1 to ensure all searching tasks eventually being successful. In each test, the service manager randomly picks three resource types and then emits the search messages to the multicast address. In the tests with efficiency-first policy, the elapsed time from emitting the search message to receiving the first response message sent from the matched Web Thing is recorded. On the contrary, in the tests with quality-first policy, the elapsed time from emitting the search message to receiving all response message sent from the matched Web Things is recorded. After that, we increase the number of managed Web Things and re-perform the tests. The experiments were performed 1000 times under each configuration and the average elapsed time is recorded.

**Figure 8:**
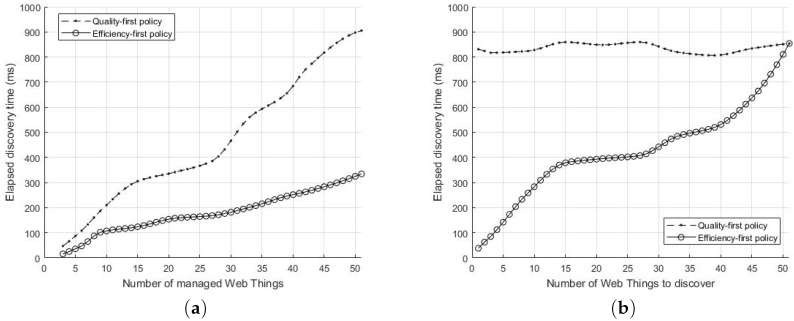
Elapsed time for performing service discovery based on ROSA. (**a**) Discovering 3 Web Things (γ=3) with different number of managed Web Things (*m*); (**b**) Discovering different number of Web Things (γ) while the total number of managed Web Things is fixed at 51 (m=51) .

Figure 8a,b show the elapsed times for searching three Web Things with specified types under a different number of Web Things (*m*) and the different number of searching resource types (γ), respectively. The overall service discovery process of ROSA involves several steps: (1) Emitting a proactive search message to the multicast address; (2) Waiting for the responses of Web Things; (3) Based on the URI provided in the response header, fetch the WTM document of the target Web Thing. The results indicate that under the quality-first policy, the average elapsed time increased as *m* increased. Similarly, if the efficiency-first policy was used, the elapsed time slightly increased as *m* increased. As expected, the elapsed time of efficiency-first policy is always shorter than that of quality-first policy as the quality-first policy requires the service manager to wait for all responses so that the service manager can pick the best ones. Similar results can be observed in the experiments shown in Figure 8b. The reason is that the higher γ, the more time it takes to find all required Web Things. It is also worthy to note that the elapsed time for the quality-first policy in Figure 8b is quite stable which is also reasonable because the total number of Web Things were fixed in the experiments (i.e., m=51). In the experiments, the overall elapsed time is typically less than 1000 ms.

**Figure 9:**
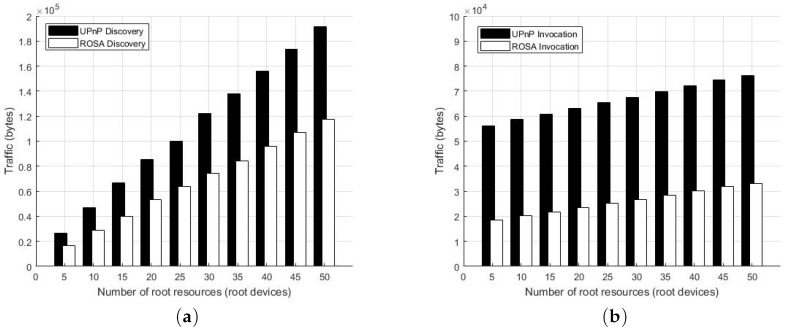
Network traffic generated by UPnP and ROSA. (**a**) Discovery; (**b**) Control.

The second set of experiments is to find out the traffic of UPnP and ROSA. Figure 9a shows the traffic (in bytes) caused by ROSA and UPnP for searching three Web Things with specified types under a different number of Web Things. The network traffic was measured using Wireshark, a free packet analyzer, installed on one of the PCs attached to the router. Figure 9a reveals that the traffic increased linearly as the number of Web Things grows and that UPnP discovery caused more traffic than ROSA discovery. Figure 9b shows the traffic (in bytes) caused by ROSA and UPnP for invoking services (i.e., controlling things) deployed on the different number of Web Things. In the experiments, each Web Thing has four service variables, and each of these variables was queried and changed for one time. Again, the results show that the traffic of service invocation increased linearly as the number of Web Things grows and that UPnP control caused more traffic than ROSA control. Note that both UPnP and ROSA generated a fixed amounts of background traffic.

### 6.4. Costs and Limitations

In this section, we discuss the costs and limitations of ROSA as well as the issues that are out of the scope of this work.

#### 6.4.1. Compatibility with LLN

First, like UPnP, ROSA is primarily designed for IP-based home network, and it works well when the underlying MAC and physical layer protocols being Ethernet or WiFi. However, there can be performance issues when it comes to 6LowPAN over LLN. For example, both UPnP and ROSA perform presence management and service discovery based on IP multicast. Thus, the service management traffic can easily flood the LLN with multicast packets. What is more, the fact that MTU of LLN is much lower than that of traditional IP network makes the traffic in LLN heavier. The is the reason why CoAP, instead of HTTP, is usually used with 6LoWPAN over LLN. Accordingly, to work well with the Web Things in the 6LowPAN over LLN in a smart home, compact and semi-centralized service discovery architecture is preferred so that multicast can be reduced to some extent [50]. In a large-scale outdoor space, the P2P-based gossip-style approaches [81] can be more desirable. For a diversified home network like Figure 1, which consists of a WiFi network, an Ethernet network, an IEEE 802.15.4 based wireless sensor network, and a home automation network with a proprietary protocol, a gateway that serves as a proxy to the non-IP network is required. In this case, the gateways are also Web Things that are managed by ROSA. As noted in [48], this integration strategy is common in building WoT systems and is also known as the gateway integration pattern.

#### 6.4.2. Node Counts

In the experiments, we focus on observing the network overhead instead of the computation power. Thus, instead of deploying them on specific embedded systems, the Web Things are evenly distributed over PCs connected to the same LAN. As SSDP in UPnP (and thus the ROSA discovery schemes) relies on HTTP-MU (HTTP over multicast UDP), depending on the number of Web Things and the PA period (i.e., heartbeat), there is a threshold where flooding occurs when the traffic is heavier than this limit. Also, when the traffic is heavy, UDP is very likely to lose packets, causing a sudden drop of performance of the network. As a result, when the number of nodes exceeds the flooding threshold, then the results of the experiments do not make much sense as in this situation the packets lost constantly and therefore both UPnP and ROSA do not work at all. Given these discussions, we perform the experiments with the number of devices up to 50 nodes. With the gateway integration pattern, theoretically, there can be 300–500 nodes operate concurrency in a smart home with the star of stars topology, but, only 50 nodes are directly managed by ROSA.

#### 6.4.3. Security and Privacy

ROSA does not include new security and privacy mechanisms. In practice, ROSA deals with security and privacy issues such as data confidentiality, data integrity, non-repudiation, authentication and authorization using the mechanism used in UPnP and HTTP. Since ROSA is designed based on HTTP, it can ensure data confidentiality based on SSL/TLS. In fact, the UPnP security profile [82] adopts this approach. However, in case that the Web Things have limited computing resources such as network bandwidth, CPU, and memory, Symmetric-Key Encryption mechanisms such as DES (Data Encryption Standard)/Triple DES [83,84] or AES [85] can be more feasible. For example, ZigBee network uses AES encryption with 128-bit key length [27]. The major challenge of using a Symmetric-Key Encryption is how to transmit the secret key over an unsecured network. In the residential mode, ZigBee chooses to ignore the potential vulnerability. One possible solution is to distribute the secret key using Asymmetric-Key Encryptions. Data integrity and non-repudiation in ROSA can be realized by using the message digest and digital signature mechanisms. To ensure data integrity and non-repudiation in ROSA, the client first obtains a message digest by using hash algorithms such as SHA (Secure Hash Algorithm) [86]. The digital signature can be generated by encrypting the message digest using the private key of the sending node, which is then placed in the header of the message before it is sent. After the Web Thing receives the message, it first obtains a message digest from the decrypted message and then compares it with the one obtained by decrypting the digital signature. Finally, the receiving Web Thing can ensure that the message is sent from a specific client if the message digests are identical. To support authentication and authorization, each Web Thing has to be enhanced according to the UPnP Security Ceremonies [82]. Specifically, every Web Thing has an additional Device Security Service which supports authentication and authorization functionalities. The security policies have to be pre-configured by users in the user interfaces. The costs of employing the security and privacy mechanisms are: (1) the efficiency of ROSA will be degraded; and (2) setting up security policies, authentication, and authorization is labor intensive and may cause inconveniences to users.

#### 6.4.4. Interoperability

It can be observed from the protocol stack that a ROSA client cannot access UPnP Devices and vice versa. However, ROSA and UPnP can co-exist in the same LAN without any interference. The reason is that the UPnP Specification requires a UPnP device to check the HTTP header and to ignore messages that are not valid UPnP messages. One possible approach to making UPnP and ROSA interoperable is to use the Gateway Integration Pattern [48]. For example, we can construct a Web Thing to serve as the gateway to UPnP network so that it acts as a proxy and translates the messages between two networks.

#### 6.4.5. Service Composition

Like UPnP, ROSA can support service composition, but ROSA itself does not perform service composition. The issues of service composition and service maintenance are out of the scope of this paper. In [87,88], we proposed a robust service composition and maintenance technique on top of UPnP and a set of auto-unifiable preference expressions that facilitates automatic service composition, respectively. These approaches can be adopted to ROSA with minor modifications on the service manager and the descriptions of Web Things. Matching of services is a key issue in service composition. Deciding if two services are equivalent is not an easy task and is also out of the scope of this paper. WS-* style Web services tend to determine the equivalence based on the signatures of remote functions, which only ensures that two services are syntactically equal. Semantic Web and Linked Data technologies can be used to compare if two Web Things are semantically equivalent and to decouple physical devices from the semantical descriptions of functions.

## 7. Conclusions

The WoT concepts and applications are becoming popular in recent years because low-cost embedded systems are now able to serve as HTTP servers and process HTTP requests. This paper presents a set of service management schemes for managing Web Things in a smart home called ROSA. UPnP, a popular standard for service management in the consumer market, is chosen as the baseline scheme. Firstly, a set of formal notations for describing interactions between Web clients and Web Things is used for specifying both UPnP and ROSA service management schemes. Based on these formal expressions, we evaluate the Web Friendliness of UPnP and ROSA based on RMM. Then, to verify the feasibility of ROSA, an air conditioning service is realized based on ROSA-capable Web Things. In the process of constructing the scenario, the notation is helpful, in a language and platform independent way, for Web Thing developers when implementing ROSA schemes. Finally, experiments that test the performance and traffics overhead caused by ROSA is conducted. The results show that when compares to UPnP, ROSA can realize same management functionalities with less overhead. The main limitation of ROSA is that it can not work well with 6LoWPAN because it employs multicast-based schemes for presence management and service discovery. As the nature of underlying networks and 6LoWPAN is usually quite different than that of the typical Ethernet-based network, we are currently studying and devising a set of CoAP-based service management schemes for 6LoWPAN nodes so that the service management schemes can work more efficiently. Besides, we are also working on a web-based tool for mashing-up the resources on top of ROSA, so that we can take one step further toward the vision of end user programming at home.

## Abbreviations

The following abbreviations are used in this manuscript:

6LoWPAN	IPv6 over Low-Power Wireless Personal Area Networks
A-URI	Abstract URI
C-URI	Concrete URI
GENA	General Event Notification Architecture
HATEOAS	Hypermedia As The Engine Of Application State
HTTPMU	HTTP over UDP Multicast
HTTPU	HTTP over UDP
IoT	Internet of Things
PA/LA	Presence Announcement/Leave Announcement
REST	Representational State Transfer
ROSA	Resource Oriented Service Administration
RMM	Richardson Maturity Model
SSDP	Simple Service Discovery Protocol
UPnP	Universal Plug and Play
WoT	Web of Things
WTM	Web Things Model
